# Diabetes Mellitus Increases Severity of Thrombocytopenia in Dengue-Infected Patients

**DOI:** 10.3390/ijms16023820

**Published:** 2015-02-10

**Authors:** Chung-Yuan Chen, Mei-Yueh Lee, Kun-Der Lin, Wei-Hao Hsu, Yaun-Jinn Lee, Pi-Jung Hsiao, Shyi-Jang Shin

**Affiliations:** 1 Chin Pin Clinic, Kaohsiung 807, Taiwan; E-Mail: wecaredm223@yahoo.com.tw; 2 Department of Internal Medicine, Kaohsiung Municipal Hsiao-Kang Hospital, Kaohsiung 812, Taiwan; E-Mails: lovellelee@hotmail.com (M.-Y.L.); my345677@yahoo.com.tw (W.-H.H.); 3 Division of Endocrinology and Metabolism, Department of Internal Medicine, Kaohsiung Medical University Hospital, Kaohsiung 807, Taiwan; E-Mail: 890073@kmuh.org.tw (K.-D.L.); pjhsiao@cc.kmu.edu.tw (P.-J.H.); 4 Lee’s Clinic, Ping Tung 900, Taiwan; E-Mail: leesclinic@leesclinic.org; 5 Center for Lipid and Glycomedicine Research, Kaohsiung Medical University, Kaohsiung 807, Taiwan

**Keywords:** thrombocytopenia, diabetes, dengue, hypoalbuminemia

## Abstract

Background: Diabetes mellitus is known to exacerbate bacterial infection, but its effect on the severity of viral infection has not been well studied. The severity of thrombocytopenia is an indicator of the severity of dengue virus infection. We investigated whether diabetes is associated with thrombocytopenia in dengue-infected patients. Methods: We studied clinical characteristics of 644 patients with dengue infection at a university hospital during the epidemic on 1 June 2002 to 31 December 2002 in Taiwan. Platelet counts and biochemical data were compared between patients with and without diabetes. Potential risk factors associated with thrombocytopenia were explored using regression analyses. Results: Dengue-infected patients with diabetes had lower platelet counts than patients without diabetes during the first three days (54.54 ± 51.69 *vs.* 86.58 ± 63.4 (*p* ≤ 0.001), 43.98 ± 44.09 *vs.* 64.52 ± 45.06 (*p* = 0.002), 43.86 ± 35.75 *vs.* 62.72 ± 51.2 (*p* = 0.012)). Diabetes mellitus, death, dengue shock syndrome (DSS) and dengue hemorrhagic fever (DHF) and increased glutamic-pyruvate transaminase (GPT) levels were significantly associated with lower platelet counts during the first day of hospitalization for dengue fever with regression β of −13.981 (95% confidence interval (CI) −27.587, −0.374), −26.847 (95% CI −37.562, −16.132), and 0.054 (95% CI 0.015, 0.094) respectively. Older age, hypoalbuminemia, and hypertriglyceridemia were independently correlated with thrombocytopenia in dengue patients with or without diabetes with regression β of −2.947 (*p* = 0.004), 2.801 (*p* = 0.005), and −3.568 (*p* ≤ 0.001), respectively. Diabetic patients with dengue had a higher rate of dengue hemorrhagic fever (DHF)/dengue shock syndrome (DSS) than non-diabetic patients. They also had lower blood albumin, were older, and higher triglyceride levels. Older age, hypoalbuminemia, and hypertriglyceridemia were independently correlated with thrombocytopenia in dengue patients. Conclusions: Dengue patients with diabetes tended to have more severe thrombocytopenia and were more likely to have DHF/DSS. Older age, hypoalbuminemia, and hypertriglyceridemia were independently associated with more severe thrombocytopenia in dengue patients.

## 1. Introduction

Diabetes mellitus is known to increase a person’s susceptibility to infection [1]. It has been found to have detrimental effects on the immune system, including decreased chemotaxis, leukocyte adherence, and phagocytosis [2,3]. In fact, certain rare infections are reported to occur more frequently in diabetic patients, though many more commonly encountered infectious diseases have not still formally been evaluated to be associated with diabetes [1,4]. However, individuals with diabetes tend to have higher risk for morbidity and mortality from commonly found infections [5,6,7,8]. These studies have focused on bacterial infections, and few have studied the interaction between diabetes and the severity of viral infections [9,10].

Dengue virus, a mosquito-borne human viral pathogen, has been an important public health problem in tropical and subtropical countries [11,12]. The severity of dengue infection ranges from nonspecific, self-limiting dengue fever (DF) to life-threatening dengue hemorrhagic fever (DHF) and dengue shock syndrome (DSS) [12,13]. Thrombocytopenia is one of the most important clinical features in dengue disease [13,14]. The mechanisms of thrombocytopenia include transient myelosuppression and dysfunction, consumption, and increased destruction of platelets [14,15].

For the past decades, Taiwan had a large outbreak of DF [16]. Because thrombocytopenia has been found to be more severe in patients with DHF than in those with DF [17,18,19,20], it may be a useful indicator of severity of dengue infection. In this retrospective study, we analyzed clinical data from dengue-infected patients to investigate potential link between diabetes and the severity of thrombocytopenia.

## 2. Results

A total of 644 patients diagnosed with dengue infection at Kaohsiung Medical University Hospital were studied. Sixty-five of these patients had type 2 diabetes mellitus. Twenty-eight of those with diabetes had DF, 34 had DHF/DSS, and 3 died (Table 1). The mean duration of diabetes was 8.6 ± 7.3 years. Patients with dengue infections and diabetes were older and had a higher rate of DHF/DSS.

The diabetic and non-diabetic patients did not differ significantly with regard to gender and mean BMI (Table 1). Diabetic patients had higher blood glucose levels, lower serum albumin levels, higher cholesterol and triglyceride levels, and higher systolic blood pressures on the day of admission than non-diabetic patients. They did not differ significantly in blood urea nitrogen (BUN), creatinine, glutamate oxaloacetate transaminase (GOT), glutamic-pyruvate transaminase (GPT), or total protein levels. Of the 11 patients who died, three had diabetes (two males) and eight did not (four males). The mortality rate was 4.62% in the diabetic patients and 1.38% in the non-diabetic patients, an insignificant difference.

**Table 1:** Clinical characteristics of dengue-infected patients by diabetes status on the first day of hospitalization.

Variables	DM	Non-DM	*p* Value
Number	65	579	–
Age (years)	59.51 ± 9.87	46.23 ± 18.05	<0.001
Male gender (%)	32 (49.23)	263 (45.42)	0.601
BMI (kg/m^2^)	26.2 ± 4.50	24.6 ± 4.60	0.110
Duration of diabetes (years)	8.64 ± 7.30	–	–
DHF, DSS, death (%)	37 (56.92)	196 (33.85)	<0.001
Death (%)	3/65 (4.62%)	8/579 (1.38%)	0.056
sBP (mmHg)	129.1 ± 16.6	120.4 ± 14.4	<0.001
dBP (mmHg)	78.9 ± 8.7	77.1 ± 10.4	0.226
Glucose (mmol/L)	12.04 ± 5.74	6.20 ± 1.78	<0.001
HbA1c (%)	8.62 ± 1.86	–	–
Total protein (g/L)	64.5 ± 6.9	65.0 ± 6.7	0.601
Albumin (g/L)	35.9 ± 3.8	37.2 ± 3.5	0.011
Cholesterol (mmol/L)	4.22 ± 1.20	3.69 ± 1.51	0.007
TG (mmol/L)	2.20 ± 0.82	1.63 ± 0.83	<0.001
BUN (mmol/L)	6.40 ± 1.27	5.09 ± 1.16	0.165
Creatinine (µmol/L)	122.88 ± 49.62	100.78 ± 38.12	0.404
GOT (IU/L)	326.27 ± 79.08	342.2 ± 75.45	0.969
GPT (IU/L)	215.45 ± 65.44	233.44 ± 65.16	0.992
CRP (mg/L)	27.56 ± 10.82	19.28 ± 5.66	0.069

DM = Diabetes mellitus; BMI = Body mass index; DHF = Dengue hemorrhagic fever; DSS = Dengue shock syndrome; sBP = Systolic blood pressure; dBP = Diastolic blood pressue; HbA1c = Hemoglobin A1c; TG = Triglycerides; BUN = Blood urea nitrogen; GOT = Glutamate oxaloacetate transaminase; GPT = Glutamic-pyruvate transaminase; CRP = C-reactive protein.

The most notable indicator was that diabetic patients had a lower mean platelet count than non-diabetic patients during the first three days of hospitalization. The WBC counts and hematocrit levels of the two groups did not differ significantly on days 1, 2, 3 and 7 of hospitalization (Table 2).

**Table 2:** White blood cell count, hematocrit, and platelet count of dengue-infected patients by diabetes status.

Days of Hospitalization	DM	Non-DM	*p* Value
Number	65	579	–
**WBC (×10^3^/μL)**			
1 day	4.51 ± 2.59	4.08 ± 2.49	0.197
2 days	4.51 ± 2.66	4.25 ± 3.17	0.501
3 days	5.81 ± 4.15	4.69 ± 3.09	0.072
7 days	7.28 ± 7.27	5.19 ± 2.74	0.218
**Hct (%)**			
1 day	40.51 ± 5.31	39.93 ± 5.54	0.432
2 days	39.45 ± 5.66	38.67 ± 5.08	0.296
3 days	39.20 ± 4.98	38.57 ± 4.94	0.405
7 days	36.64 ± 4.82	36.46 ± 4.95	0.875
**PLT (×10^3^/μL)**			
1 day	54.54 ± 51.69	86.58 ± 63.4	<0.001
2 days	43.98 ± 44.09	64.52 ± 45.06	0.002
3 days	43.86 ± 35.75	62.72 ± 51.21	0.012
7 days	102.35 ± 78.96	84.46 ± 54.18	0.338

DM = Diabetes mellitus; WBC = White blood cell; Hct = Hematocrit; PLT = Platelet.

After adjusting for age, gender, body mass index, glucose, albumin, creatinine, SGOT, CRP, cholesterol, triglycerides, systolic blood pressure and diastolic blood pressure, it could be observed that diabetes mellitus, death, DSS and DHF and increased GPT levels significantly lowered the platelet counts during the first day of hospitalization for dengue fever with regression β of −13.981 (95% confidence interval (CI) −27.587, −0.374), −26.847 (95% CI −37.562, −16.132), and 0.054 (95% CI 0.015, 0.094), respectively (Table 3).

**Table 3:** Multivariate stepwise linear regression analysis for the correlation of platelet counts with variables on the first day of hospitalization for dengue fever.

Variables	Multiple		*p* Value
	Regression β	95% CI	
Death, DSS, DHF	−26.847	−37.562, −16.132	<0.001
GPT	0.054	0.015, 0.094	0.007
DM	−13.981	−27.587, −0.374	0.044
HbA1c	4.454	−1.109, 10.017	0.113

Covariate included age, gender, body mass index, glucose, albumin, creatinine, glutamate oxaloacetate transaminase, C-reactive protein, cholesterol, triglycerides, systolic blood pressure, diastolic blood pressure. Gender is represented by means of dummy variable. DM = Diabetes mellitus; DHF = Dengue hemorrhagic fever; DSS = Dengue shock syndrome; GPT = Glutamic-pyruvate transaminase; HbA1c = Heamoglobin A1c.

As can be seen in Table 4, on day one, the Pearson correlation between platelet count and the covariates of the diabetic and non-diabetic patients differed significantly. A lower platelet count was significantly correlated with older age, higher systolic blood pressure, higher triglyceride level, lower serum albumin level, and lower total cholesterol level. Multiple linear regression analysis further revealed that platelet count was independently correlated with age, triglyceride, albumin and cholesterol levels.

**Table 4:** Linear regression analysis for the correlation of platelet counts with continuous variables on the first day of hospitalization for dengue fever including diabetics and non-diabetics.

Variables	Spearman		Multiple	
	γ	*p* Value	â	*p* Value
Age	−0.300	<0.001	−2.947	0.004
sBP (mmHg)	−0.109	0.026	−0.671	0.503
Glucose (mmol/L)	−0.090	0.055	−0.671	0.820
Albumin (g/L)	0.316	<0.001	2.801	0.005
Cholesterol (mmol/L)	0.211	<0.001	3.736	<0.001
Triglyceride (mmol/L)	−0.239	<0.001	−3.568	<0.001

sBP = systolic blood pressure.

In Table 5 and Table 6, four models of linear regression analysis were created to assess the correlation of platelet count with serum albumin and triglyceride concentrations while adjusting continuous covariates. All models showed that decreased platelet count was independently correlated with decreased albumin concentrations. Decreased platelet count was independently correlated with increased triglyceride levels.

**Table 5:** Standardized regression coefficients for the correlation of platelet count with serum albumin concentrations after adjusting for continuous covariates on the first day of hospitalization for dengue fever including diabetics and non-diabetics.

Models	Standardized â	*p* Value
Without adjust	0.316	<0.001
Adjusted for age, gender, glucose	0.229	<0.001
Adjusted for age, gender, glucose, sBP, dBP, Chol, TG	0.215	0.009
Adjusted for age, gender, glucose, sBP, dBP, Chol, TG, total protein, BMI, BUN, creatinine, GOT, GPT, CRP	0.213	0.014

Gender is represented by means of dummy variable. sBP = Systolic blood pressure; dBP = Diastolic blood pressure; Chol = Cholesterol; TG = Triglycerides; BMI = Body mass index; BUN = Blood urea nitrogen; GOT = Glutamate oxaloacetate transaminase; GPT = Glutamic-pyruvate transaminase; CRP = C-reactive protein.

**Table 6:** Standardized regression coefficients for the correlation of platelet count with serum triglyceride concentrations after adjusting for continuous covariates on the first day of hospitalization for dengue fever including diabetics and non-diabetics.

Models	Standardized â	*p* Value
Without adjust	−0.239	<0.001
Adjusted for age, gender, glucose	−0.240	<0.001
Adjusted for age, gender, glucose, sBP, dBP, Chol, total protein, albumin	−0.207	<0.001
Adjusted for age, gender, glucose, sBP, dBP, Chol, total protein, albumin, BMI, BUN, creatinine, GOT, GPT, CRP	−0.367	<0.001

Gender is represented by means of dummy variable. sBP = Systolic blood pressure; dBP = Diastolic blood pressure; Chol = Cholesterol; BMI = Body mass index; BUN = Blood urea nitrogen; GOT = Glutamate oxaloacetate transaminase; GPT = Glutamic-pyruvate transaminase; CRP = C-reactive protein.

## 3. Discussion

To our knowledge, this is the first study to report a correlation between diabetes and increased severity of thrombocytopenia in dengue infection. A greater proportion of patients with diabetes had the more severe forms of dengue infection, DHF/DSS, than those without. It has been found that uncontrolled diabetes can increase the mortality and morbidity caused by serious infections, lowering the host’s defense mechanisms by impairing phagocytosis, intracellular killing, and chemostaxis of ploymorphonuclear leukocytes [2,3]. It has also been found to be an independent predictor of pleural effusion and mortality in patients with community-acquired pneumonia [7]. Bertoni *et al.*, basing their findings on a national health and nutrition survey of 9280 adults, reported that diabetic adults were at greater risk for infection-related mortality [5]. However, most of these studies were conducted on bacterial infections; few have examined the morbidity and mortality of viral infection in diabetic patients [9,10].

Patients with DHF have been found to have significantly lower platelet counts than those with the less severe DF [17,18,19,20]. Therefore, it is reasonable to infer that the degree of thrombocytopenia could be used as an indicator of the severity of dengue infection. In the current study, patients with diabetes had a higher proportion of DHF/DSS and significantly lower platelet counts, suggesting that diabetes may predispose them to a more severe dengue infection. However, the pathophysiology behind diabetes leading to a DHF outcome is not well understood yet, even though numerous studies had suggested that diabetes mellitus can result in immune and endothelial dysfunction [21,22,23,24,25].

Studies on bacterial infections have suggested that the excess risks associated with diabetes may be mediated by advanced age, cardiovascular disease, obesity, chronic renal insufficiency, hypoalbuminemia or hypocholesterolemia [7]. Pang *et al.* have also shown that diabetes with hypertension increased the risk of DHF [26]. In this study, we found that age, systolic blood pressure, plasma glucose level, serum cholesterol level, and triglyceride level were higher and serum albumin levels were lower in patients with diabetes than in patients without diabetes. These differences might be linked to a greater degree of thrombocytopenia in patients with diabetes. Simple linear regression analysis showed that age, systolic blood pressure, and triglyceride level to be negatively correlated with platelet count, while serum albumin and cholesterol levels were positively correlated with platelet count in all of the dengue patients. Furthermore, multiple linear regression analysis revealed that advanced age, higher triglyceride levels, lower albumin levels, and lower cholesterol levels were independently associated with thrombocytopenia. In bacterial infections, advanced age is known to be related to severity, prognosis, and mortality [7,27]. In our study of this viral infection, there was a correlation between older age and greater severity of thrombocytopenia in dengue patients.

In acutely ill patients, hypoalbuminemia has been strongly associated with poor clinical outcomes, including morbidity, mortality, and prolonged hospital stays [28,29] and it has been advocated that albumin levels be monitored in these patients [28]. Growing attention has been paid to the inflammatory processes that induce hypoalbuminuria by increasing vascular permeability and the inflammatory mediators that promote albumin leakage into extravascular space. Our study has also linked decreased albumin concentration and greater severity of thrombocytopenia (*i.e.*, lower platelet count).

Acute microbial infections have a remarkable effect on lipid metabolism [30]. Infections caused by Gram-negative bacteria have been shown to affect triglyceride and high-density lipoprotein (HDL) cholesterol levels. Administration of lipopolysaccharides (LPS) decreases total cholesterol and HDL cholesterol levels [31,32]. Administration of tumor necrosis factor (TNF) or interleukin-1results in a rapid elevation of serum triglyceride levels [33,34]. These infection-induced alterations in lipid metabolisms are considered to be one part of the acute-phase immune response. In fact, low cholesterol levels were found to be a predictor of nonsocial infection in hospitalized patients and associated with the severity of pneumonia [29]. We found a correlation between lower total cholesterol level and lower platelet count in overall dengue patients. However, while dengue patients with diabetes had significantly higher total cholesterol concentrations than non-diabetic patients, their platelet counts were lower, suggesting that total cholesterol level may not be related to the development of thrombocytopenia in diabetic patients with dengue infection.

Total triglyceride level was inversely correlated with platelet count in overall dengue patients. Multiple linear regression analysis revealed an independent association between triglyceride concentration and platelet count. Diabetic patients with dengue infection had a significantly higher triglyceride concentration and lower mean platelet counts than those without diabetes. These findings suggest that increased serum triglyceride levels may be correlated to the severity of thrombocytopenia in diabetic patients. The relationship between platelets and triglyceride concentrations in dengue infection requires further investigation.

The absence of HbA1c data in dengue-infected patients without diabetes is one limitation of our study. Instead of HbA1c, we analyzed the correlation between plasma glucose values on the first day of hospitalization and patients’ platelet counts on the first day of hospitalization. Plasma glucose was measured at either an emergency room or outpatient department. The result of such a plasma glucose study in those with acute conditions may reflect transient and/or stress-related hyperglycemia. Some studies have suggested that hyperglycemia at hospital admission cannot predict mortality or morbidity in critically ill patients [35]. Indeed, our study found no significant correlation between plasma glucose levels and platelet counts in dengue patients.

## 4. Materials and Methods

In the dengue epidemic in Taiwan, a person was suspected being infected with dengue virus if he or she had a body temperature higher than 38 °C and one of the following symptoms and signs: headache, retro-orbital pain, myalgia, arthralgia, rash, and pruritis. Blood samples from all patients with suspected dengue infection were tested in the laboratory to confirm the diagnosis. A dengue infection was confirmed if any of the following criteria were met: (1) Positive dengue virus isolation; (2) Positive detection of dengue virus RNA by reverse transcriptase chain reaction; (3) A four-fold increase of dengue virus-specific immunoglobulin M(IgM) or IgG antibody in paired serum samples after cross-reactions to Japanese encephalitis had been excluded; or (4) Positive identification of dengue virus-specific IgM and IgG antibody in a single serum sample after cross-reactions to Japanese encephalitis had been excluded.

This study included 644 patients with confirmed dengue infections at Kaohsiung Medical University Hospital in Kaohsiung, Taiwan during the period between on 1 June 2002 to 31 December 2002. This study was approved by the Institutional Review Board with document number KMUH-IRB-20140274. The diagnosis of DHF was made based on whether the patient had thrombocytopenia (platelet count less than 100,000/µL) and evidence of hemorrhage and plasma leakage such as hemoconcentration, hypoalbuminuria, hypoproteinemia, pleural effusion, or ascites. Hemoconcentration was defined as an increase of hematocrit by 20% or more from baseline or a decrease by 20% or more after hydration; it was calculated as the difference between maximum and minimal hematocrit values divided by the minimal value. Pleural effusion or ascites was assessed by X-ray, ultrasound, or computerized axial tomography. Hypoalbuminuria was defined as a serum albumin level less than 3 g/dL. Dengue shock syndrome (DSS) was diagnosed if the patient presented hypotension or narrowing of the pulse pressure to less than 20 mmHg with clinical signs of shock. Hypotension was defined as systolic blood pressure below 80 mmHg.

We collected the following data for each patient: age, sex, medical history (diabetes and its duration; hypertension), height, and weight. Blood samples were obtained from these patients on the first day (day 1) and on days 2, 3, and 7. Hematological biochemical analysis included white blood cell (WBC) count, hematocrit, platelet count, blood glucose, blood urea nitrogen (BUN), serum creatinine, liver function tests (GOT and GPT), cholesterol, triglyceride, serum albumin, total protein, c-reactive protein (CRP), and hemoglobin A1c (HbA1c for diabetic patients only). The blood pressure of all patients was measured and included in the analyses. Diabetes was recorded if a patient had a documented history of the disease and was receiving anti-diabetic drug treatment.

All data were expressed as means ± standard deviation (SD). The statistical operations were performed using SPSS version 12.0 (SPSS Inc., Chicago, IL, USA). Student’s *t* test and Chi-square test were used to compare the variables between diabetic and non-diabetic patients. Spearman correlations and multiple linear regression analysis were used to analyze platelet counts and variables significantly different between diabetic and non diabetic patients. Linear regression models were created using serum albumin (Table 5) and triglyceride (Table 6) as dependent variables and platelet levels as an independent variable. Four models were created: (1) Without adjustment; (2) Adjusted for age, sex, and plasma glucose level; (3) Adjusted for age, sex, and variables that are significantly different between diabetic and non-diabetic patients; (4) Adjusted for all covariates: age, sex, plasma glucose level, blood pressure, cholesterol, triglyceride (or albumin), total protein, body mass index (BMI), BUN, creatinine, GOT, GPT, and CRP. Gender is represented by means of a dummy variable, which are constructed in order to include non-quantitative factors in a regression model. These factors can distinguish two more categories, in such a way that each dummy variable takes one value for the category we consider, and zero value for the rest of categories. A *p* value <0.05 was considered significant.

## 5. Conclusions

In conclusion, dengue-infected patients with diabetes had more severe thrombocytopenia and were more likely to have DHF/DSS. Hypoalbuminemia, hypertriglyceridemia, and older age were associated with more severe thrombocytopenia in patients with dengue infection.
